# cnvScan: a CNV screening and annotation tool to improve the clinical utility of computational CNV prediction from exome sequencing data

**DOI:** 10.1186/s12864-016-2374-2

**Published:** 2016-01-14

**Authors:** Pubudu Saneth Samarakoon, Hanne Sørmo Sorte, Asbjørg Stray-Pedersen, Olaug Kristin Rødningen, Torbjørn Rognes, Robert Lyle

**Affiliations:** Department of Medical Genetics, Oslo University Hospital and University of Oslo, Oslo, Norway; Norwegian National Newborn Screening, Oslo University Hospital, Oslo, Norway; Center for Human Immunobiology/Section of Immunology, Allergy, and Rheumatology, Texas Children’s Hospital, Houston, TX USA; Baylor-Hopkins Center for Mendelian Genomics of the Department of Molecular and Human Genetics, Baylor College of Medicine, Houston, TX USA; Department of Informatics, University of Oslo, Oslo, Norway; Department of Microbiology, Oslo University Hospital, Oslo, Norway

**Keywords:** CNV, Exome, Mutation detection

## Abstract

**Background:**

With advances in next generation sequencing technology and analysis methods, single nucleotide variants (SNVs) and indels can be detected with high sensitivity and specificity in exome sequencing data. Recent studies have demonstrated the ability to detect disease-causing copy number variants (CNVs) in exome sequencing data. However, exonic CNV prediction programs have shown high false positive CNV counts, which is the major limiting factor for the applicability of these programs in clinical studies.

**Results:**

We have developed a tool (cnvScan) to improve the clinical utility of computational CNV prediction in exome data. cnvScan can accept input from any CNV prediction program. cnvScan consists of two steps: CNV screening and CNV annotation. CNV screening evaluates CNV prediction using quality scores and refines this using an in-house CNV database, which greatly reduces the false positive rate. The annotation step provides functionally and clinically relevant information using multiple source datasets.

We assessed the performance of cnvScan on CNV predictions from five different prediction programs using 64 exomes from Primary Immunodeficiency (PIDD) patients, and identified PIDD-causing CNVs in three individuals from two different families.

**Conclusions:**

In summary, cnvScan reduces the time and effort required to detect disease-causing CNVs by reducing the false positive count and providing annotation. This improves the clinical utility of CNV detection in exome data.

**Electronic supplementary material:**

The online version of this article (doi:10.1186/s12864-016-2374-2) contains supplementary material, which is available to authorized users.

## Background

With advances in next generation sequencing technology and analysis methods, single nucleotide variants (SNVs) and indels can be detected with high sensitivity and specificity in exome sequencing data [1, 2]. While recent studies have demonstrated the ability to detect disease-causing copy number variants (CNVs) [3], exonic CNV prediction programs have shown high false positive CNV counts [4]. This high false positive count is the major limiting factor for the applicability of these programs in clinical studies. Here we report a tool (cnvScan) which considerably improves the clinical utility of computational CNV prediction by reducing the false positive count and providing clinically relevant annotation.

cnvScan enables users to evaluate CNVs predicted from any program and provides robust CNV quality assessment to reduce the false positive count. As a comparison, the false positive count of SNV prediction was reduced with the availability of variant quality assessment and recalibration methods introduced by programs like GATK toolkit [5]. While commonly used CNV prediction programs (ExomeCopy [6], ExomeDepth [7], ExCopyDepth [4], CoNIFER [8] and XHMM [9]) calculate CNV quality scores providing statistical support for the prediction, researchers have not yet studied how CNV quality scores can be effectively used to filter false positive CNVs.

When considering techniques used to improve variant discovery in exome sequencing, current SNV analysis pipelines use in-house SNV databases to filter out variants due to technical artifacts and population-specific variants. In order to provide similar methods for CNV analyses, we developed a novel method using an in-house CNV database to further evaluate the quality of CNV calls.

In addition, existing CNV analysis tools such as ANNOVAR [10], VEP [11], CNVAnnotator [12] and DeAnnCNV [13] do not assess the quality scores reported by CNV prediction programs or provide the broadest range of clinically relevant data. For example, different annotation programs use different sets of source datasets when annotating CNVs [10–12] and do not use recent data sets such as the development disorder annotations from DECIPHER (DDD) [14] or high quality manually curated CNVs from the database of genomic variants (DGV) [15]). With cnvScan, we have created a central resource combining multiple different datasets to provide annotation of high quality CNVs.

To assess the clinical utility of cnvScan, we used 64 exomes from primary immunodeficiency (PIDD) patients. cnvScan greatly reduces false positive CNVs and enabled the identification of three high-quality rare CNVs in two families. Both of these CNVs were confirmed as PIDD-causing variants. cnvScan thus provides both robust CNV quality assessment and a broad range of functionally and clinically relevant information for each CNV.

## Results and discussion

As input, cnvScan can use a CNV results file from any prediction program. cnvScan then uses a two-step approach to improve the functional and clinical interpretation of computationally predicted CNVs: CNV screening and CNV annotation (Additional file 1: Figure S1).

### CNV screening

In order to generate a set of input files to test our program, we performed computational CNV prediction on exomes from 17 patients with primary immunodeficiency (PIDD) using ExomeCopy [6], ExCopyDepth [4], ExomeDepth [7], CoNIFER [8] and XHMM [9] (Methods).

CNV prediction programs calculate quality scores which provide statistical support for the predictions (Additional file 1: Table S1). But how quality scores can be used as an effective parameter when evaluating the quality of computational CNV predictions has not been tested.

The prediction programs assessed in our study employ a coverage-based approach to call CNVs. Therefore the quality of the CNV calls will be affected by factors that influence the coverage of exonic regions. For example, genomic features such as GC % can affect coverage distribution and repeat content can affect the mapping quality of aligned reads. Prediction programs model these features using different statistical approaches [16] and CNV quality scores are assigned. Therefore, we studied CNVs from all the prediction programs to test how coverage and genomic features affect quality scores.

Analysis showed that there is no strong correlation between quality scores and CNV length, GC %, repeat length and mean coverage (Additional file 1: Figure S2–S5). This indicated that the quality score is a stable measure which is less sensitive to coverage or genomic features. For example, shorter (1 kb) and longer (over 100 kb) CNVs were observed for the entire spectrum of quality scores reported (correlation coefficients = 0.00–0.23). When considering repeat content and quality scores, CoNIFER showed low repeat content for high quality CNVs. However, all the other programs showed shorter (10 bp-1 kb) and longer (over 10 kb) repeat content for all the reported quality scores.

Since this initial analysis suggested that quality scores are stable across different genomic features, we wanted to further investigate how the quality score could be used to reduce the false positive (FP) count. As a first step, we studied the relationship between the quality score and the false discovery rate (FDR). In order to calculate the FDR, we derived a set of true positive (TP) and FP CNVs by comparing CNV calls from exome sequencing and exon-focussed aCGH experiments (exaCGH [4]) from 17 PIDD patients. Next we used these TP and FP CNVs to further examine the quality scores of the programs (Methods).

**Fig. 1:**
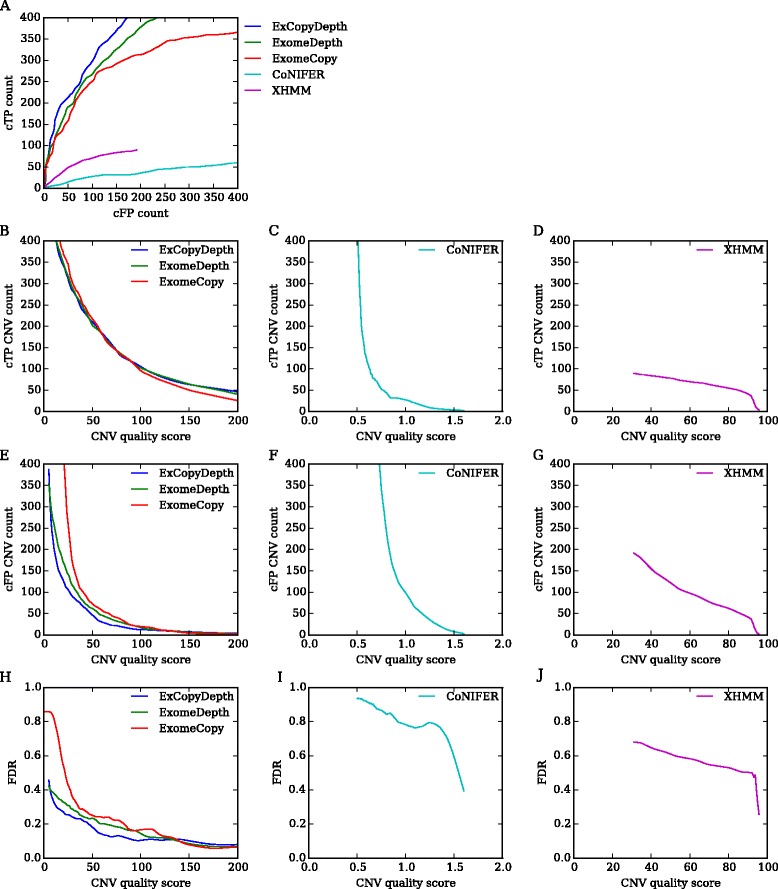
Cumulative TP (cTP) count and cumulative FP (cFP) count distribution for five programs tested in the study. **a** cTP count vs cFP count for each quality score of the prediction program. **b**, **c**, **d** cTP count distribution vs CNV quality score. **e**, **f**, **g** cFP count distribution vs CNV quality score. **h**, **i**, **j** False Discovery Rate (FDR) vs CNV quality score for five programs. FDR: False positive CNVs/(True positive CNVs + False positive CNVs). All the programs showed a decrease in FDR with increasing quality score (Pearson correlation coefficients (r) - ExomeCopy: *r* = −0.49, *p* = 2.50e-21; ExCopyDepth: *r* = −0. 56, *p* = 1.44e-74; ExomeDepth: *r* = −0.64, *p* = 7.82e-105; CoNIFER: *r* = −0.63, *p* = 0.00; XHMM: *r* = −0.98, *p* = 4.34e-269). Quality scores of different prediction programs have different ranges, therefore scores are presented in different figures. CoNIFER SVD-ZRPKM values range from −3 - +3, thus absolute values are presented in Fig. 1c, f

In order to test the applicability of quality scores for CNV quality assessment, we first studied the relationship between TP CNVs, FP CNVs and quality scores of each program. Here we calculated the cumulative TP (cTP) and cumulative FP (cFP) counts (Methods) (Fig. 1). As expected, high quality scores gave higher cTP counts compared to cFP counts. ExomeCopy, ExCopyDepth and ExomeDepth showed higher cTP count compared to cFP count for any given quality score (Fig. 1b, e). CoNIFER and XHMM showed higher cFP counts (Fig. 1f, g) due to the low thresholds used when executing these programs (Methods). Moreover, a clear inverse correlation between cFP and quality score was observed (correlation coefficient of XHMM = −0.98; Fig. 1g).

We next calculated the FDR to evaluate the relationship between FP CNVs and quality scores. Fig. 1h, i, j show how FDR varies with CNV quality score. For all the programs, FDR showed a decrease with the increasing CNV quality score. This also highlighted the applicability of the quality score as an effective parameter that can reduce FP CNV counts. For example, ExCopyDepth showed a 50 % decrease in FDR at 40.6 quality score. Thus, we were able to test and confirm the importance of the quality score in reducing FP CNV count and FDR of CNV prediction.

CNV prediction programs tested in our study call CNVs based on normalized coverage data extracted from a collection of exomes (reference collection) [17]. High coverage variance across the reference collection can affect the normalization process and consequently increase FP CNVs count. Therefore overlapping false positive CNVs can be predicted from a set of samples, when exonic regions in these samples were affected by technical artifacts. With the analysis of CNV prediction quality (Fig. 1), we observed that the majority of false positives have low scores. Therefore we hypothesized that false positive CNVs predicted from multiple samples would have lower median quality scores than the median scores of true positives. To test our hypothesis, we developed a method using an in-house CNV database.

The in-house database was developed from the exomes used in the reference collection of the prediction program and consists of the location, quality score and sample ID of each predicted CNV (Detailed description of commands used to develop the database is available in the Additional file 1: Text S1).

Since prediction programs tested in our study use different statistical approaches to assign quality scores, separate databases were created for each program. These databases were then used to assess the quality of TP and FP CNVs identified from exaCGH experiments (Methods). Here, in-house databases were searched using TP and FP CNVs as queries to identify overlapping database CNVs and their counts. Database CNV count represents the number of samples in which query CNVs were predicted. If two or more database CNVs were found, the median quality score (CNVQ) of these CNVs was calculated.

**Fig. 2:**
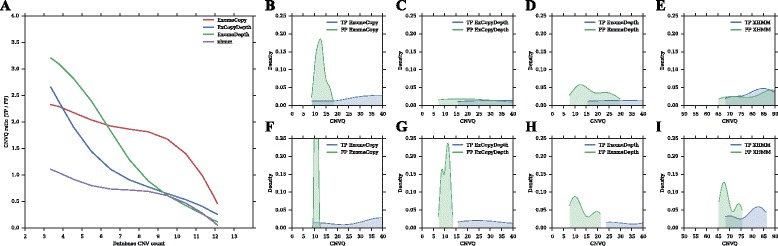
Analysis of TP CNVs and FP CNVs using in-house databases. **a** CNVQ ratio vs Database CNV count. **b**, **c**, **d**, **e** CNVQ distribution of FPs and TPs for all the database CNV counts. **f**, **g**, **h**, **i** CNVQ distribution of FPs and TPs for database CNV counts < 5. CNVQ ratio = CNVQ of TP CNVs/CNVQ of FP CNVs. Database CNV count represents the number of samples in which CNVs were found. CNV quality score is not reported by default in CoNIFER. Therefore CNVs predicted by CoNIFER were not analyzed using the in-house database

Due to the high FP count in CNV prediction, TP queries were present at low frequency and FP queries were overrepresented in the in-house database (Additional file 1: Figure S6). These overrepresented FP queries were the FP CNVs predicted from multiple exomes. Thus, we expected these FP queries to have lower CNVQs than the CNVQs of TPs. This was tested by calculating the CNVQ ratio between TP and FP CNVs (Fig. 2).

As expected, high CNVQ ratios (> 1.5) indicating low CNVQ for FPs were observed in ExomeCopy predictions. ExCopyDepth and ExomeDepth also showed higher CNVQ ratios in low database CNV counts (< 5). However, the CNVQ ratio decreases with increasing database CNV counts. For example, ExCopyDepth and ExomeDepth showed low CNVQ ratios (< 1) for higher database CNV counts (> 7). XHMM also showed low CNVQ ratios (< 1) for all the database CNV counts. ExCopyDepth, ExomeDepth and XHMM are optimized to detect rare CNVs [4, 7, 9]. Therefore these algorithms were not effective in assigning quality scores that can be used to differentiate FPs and common TPs (Additional file 1: Table S2). Hence low CNVQs or higher database CNV counts could indicate the prediction of FP or common TP CNVs.

In order to demonstrate how FPs and TPs can be differentiated using CNVQ, we compared FP and TP CNVQ distributions. The CNVQ range of FPs is lower than the CNVQ range of TPs (Fig. 2a, b, c, d, e). However there is an overlap between FP and TP distributions, indicating that FPs and low quality TP could have similar scores. This may be due to the ineffective quality score assignment in common TP CNVs (Additional file 1: Table S2). Thus, we excluded all the CNVs with high database counts (≥ 5) and compared CNVQ distributions of TPs and FPs (Fig. 2f, g, h, i). When considering low database CNV counts (< 5), FP CNVQ distributions were in the lower range of the CNVQ spectrum and TP CNVQ distributions were in the upper range. CNVQ values for all four programs can be identified to differentiate FPs and TPs. For example, in ExCopyDepth and ExomeDepth, all the FPs were lower than CNVQ ~15 and ~22. This confirmed our hypothesis and demonstrated the possibility of using CNVQ in order to differentiate TP and FP predictions.

In summary, the analysis of CNV quality scores and in-house CNV databases, suggested that cnvScan is useful in identifying TP and FP CNVs. Identifying clinically relevant CNVs remains challenging due to the number of TP CNVs identified per exome (Fig 1b). Therefore, additional information is needed to help identify CNVs with clinical significance.

### CNV annotation

In order to assess the functional effect and clinical significance of predicted CNVs, cnvScan provides an annotation step which uses data from multiple external databases (Table 1). These source datasets can be grouped into three main categories: gene and functional effect datasets, known CNVs from public databases and clinically significant datasets.

**Table 1:** Source datasets used for annotation

Source	Extracted information	Reference
Gene and functional effect datasets		
Gencode V.19	Gene name (HGNC gene symbol) Gene type Gene IDs (Ensemble) Transcript IDs (Ensemble) Exon counts (Internal to CNVs) UTRs	http://www.gencodegenes.org/releases/19.html
PhastCon	PhastCon element count PhastCon element score	http://hgdownload.soe.ucsc.edu/goldenPath/hg19/database/phastConsElements100way.txt.gz
Haploinsufficiency index	Haploinsufficiency score	http://journals.plos.org/plosgenetics/article?id=10.1371/journal.pgen.1001154
Gene intolerance	Gene intolerance score	http://chgv.org/GenicIntolerance/
Known CNVs		
Sanger high resolution CNVs	Sanger CNV count	http://www.sanger.ac.uk/science/collaboration/copy-number-variation-project
DGV	DGV CNV count Variant type Variant subtype Pubmed ID	http://dgv.tcag.ca/dgv/app/home
Curated high quality DGV	CNVs from 2 stringency levels CNV population frequencies	http://www.ncbi.nlm.nih.gov/pubmed/25645873
1000 Genomes CNVs	1000 Genomes deletion 1000 Genomes insertions	http://www.1000genomes.org/announcements/mapping-copy-number-variation-population-scale-genome-sequencing-2011-02-03
Clinically relevant information		
OMIM morbid map	OMIM disease Pubmed ID	http://www.omim.org
DECIPHER	DECIPHER development disorder genes	https://decipher.sanger.ac.uk/ddd#ddgenes
ClinVar	ClinVar disease HGVS name of the variant	http://www.ncbi.nlm.nih.gov/clinvar/

For each screened CNV, gene content (Gencode V.19) [18], level of conservation (PhastCon score) [19], predicted probability of exhibiting haploinsufficiency (haploinsufficiency score) [20] and likelihood of how well genes tolerate functional variation (genic intolerance score) [21] were annotated as functionally significant information. PhastCon, haploinsufficiency and genic intolerance scores are important to assess the biological effect of novel CNVs that are not reported in public CNV databases.

Known CNVs were identified using three datasets: Sanger high-resolution CNVs [22], 1000 Genomes CNVs [23] and Database of Genomic Variants (DGV) [24]. DGV is a continuously updated, comprehensive catalogue of CNVs. However, recent studies have identified challenges with using DGV in a clinical setting [15]. Therefore, in addition to DGV data, we extracted recently published high-quality, manually curated CNVs from DGV [15] to identify known non-disease causing CNVs. These were defined as (1) at least two subjects in one study or (2) at least two subjects each in two studies (inclusive map and stringency map [15]). Thus, these three datasets from clinically healthy populations can be used to filter out common and non-disease causing CNVs predicted from exome collection.

Clinically significant information was obtained from OMIM morbid map [25], Deciphering Developmental Disorders (DECIPHER DDD) [14] and ClinVar [26] CNVs. Thus the cnvScan annotation step provides information that can be used to assess the functional effect and the clinical significance of each predicted CNV.

### Implementation and evaluation of cnvScan

cnvScan was developed to improve the clinical utility of CNV predictions. We propose a three-stage approach to implement cnvScan and detect clinically significant CNVs: (1) CNV prediction, (2) cnvScan screening and annotation and (3) CNV filtration and disease-causing variant detection.

CNVs can be predicted from any program and the resulting CNVs used as input. Then cnvScan evaluates the CNV prediction quality and provides functional and clinical annotation. Finally, CNV filtration can be performed to detect rare, high-quality clinically relevant CNVs. Common and non-disease causing variants within the initial prediction can be filtered using cnvScan annotations (eg. Sanger high-resolution, DGV high quality and 1000 genomes data). To exclude low quality CNVs, CNV quality scores (from prediction programs) and CNVQs (from cnvScan) can be used as filtration parameters.

**Fig. 3:**
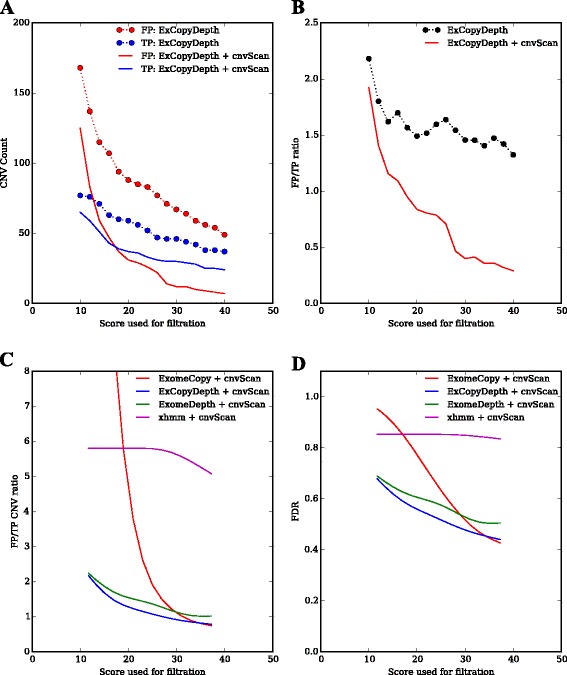
Analysis of cnvScan filtration efficiency. **a** TP and FP count vs quality score used for filtration. ExCopyDepth score: default quality score of the CNV from ExCopyDepth. cnvScan score: CNVQ from in-house database (**b**) FP/TP ratio vs quality scores used for filtration (Comparison of cnvScan efficiency using ExCopyDepth predictions). FP/TP ratio: False positive CNV count/True positive CNV count. **c** Comparison of cnvScan efficiency of four CNV prediction programs. Scores used for filtration: default CNV quality score from prediction programs and CNVQ from in-house database. **d** Comparison of the cnvScan efficiency in reducing FDR of four prediction programs (FDR of prediction programs vs cnvScan scores). CoNIFER results were not filtered using cnvScan as CNVQ is not reported in the default state

cnvScan was designed to considerably improve the time and effort required to detect disease-causing variants. To assess this, we implemented cnvScan with CNVs predicted from exomes used in the previous stage of the study (TP and FP CNVs from 17 exomes of PIDD patients). The total number of CNVs used in the cnvScan run was 1742 (ExCopyDepth predictions). Following the cnvScan run, the first filtration step identified 1004 (57.63 %) CNVs as common non-disease causing CNVs. CNVs were then filtered using CNV quality scores and CNVQs ranging from 10 to 40 (Fig. 3a). CNV quality score filtration showed decreases in FP counts compared to TP counts. Filtration on both scores (CNV quality scores and CNVQs) showed a steep decline in FP CNV counts (~170 to ~10) compared to TP counts (~80 to ~40).

We then calculated the FP/TP CNV ratio and studied how the ratio changes when implementing cnvScan over a range of quality scores. Fig. 3b indicates that CNV quality score and CNVQ can effectively filter out FP CNVs while retaining TP CNVs. For example, lower FP/TP CNV ratios (< 1) were observed for higher CNV quality score and CNVQs (> 18). Finally, we compared the filtration efficiency (CNV quality score and CNVQ filtration) for all the programs used in our study. FP/TP count ratio curves showed that cnvScan is effective in reducing FPs predicted by ExomeCopy, ExCopyDepth and ExomeDepth (Fig. 3c). A comparison of FDR of these programs (Fig. 3d) showed an improved performance for the combination of ExCopyDepth and cnvScan.

The in-house database used in the cnvScan run contains CNVs from the PIDD exome collection (*n* = 64). During cnvScan implementation, the database CNV count was not used as a filtration parameter to identify FPs since this could exclude disease-causing variants, which were predicted from multiple samples. However, the database CNV count improved the differentiation of TP and FP CNVs (Fig. 2). Thus, we wanted to test how the application of the database CNV count could improve the cnvScan filtration process (Additional file 1: Figure S7). All the programs showed low FP/TP count ratios (with low FDRs) when database CNV counts were used as an additional parameter in the cnvScan filtration. When comparing all the programs (Additional file 1: Figure S7a, b), a combination of ExomeDepth and cnvScan showed the lowest FP/TP count ratio (< 0.5) with FDR ~0.4.

In cnvScan filtration, XHMM didn’t show a decrease in FP CNVs (FP/TP ratio > 3, FDR > 0.8) for quality scores between 10 and 40 (Fig. 3c, d). Therefore XHMM predictions were filtered using scores ranging 10–100 (Additional file 1: Figure S8). When high scores (> 50) were applied in filtration, XHMM showed an improved performance with low FP/TP ratio (< 1) and FDR (~0.4). Since CoNIFER doesn’t report CNV quality scores with the default settings, cnvScan filtration efficiency of CoNIFER was not studied. However, CoNIFER predictions followed by cnvScan run are still useful to obtain functional and clinical information for predicted CNVs.

cnvScan implementation and evaluation demonstrated the ability to reduce the FP CNV count and FDR in CNV prediction. This can improve the time and effort required to detect clinically significant CNVs from computational predictions. We then applied cnvScan in a patient exome collection to test the performance of our improved method.

### Clinical utility of cnvScan

Having evaluated the efficiency of cnvScan, we wanted to study how cnvScan implementation can improve disease-causing CNV detection. We predicted CNVs using ExCopyDepth on 64 PIDD patient exomes and the resulting CNVs were assessed and annotated using cnvScan (Methods section).

Since we are interested in PIDD-causing variants, we selected only the CNVs (*n* = 769) predicted to affect known PIDD genes (*n* = 475). Next, cnvScan filtration steps were applied to detect PIDD-causing variants from this PIDD call set.

The first filtration step that remove common and non disease-causing variants, identified 210 (27.3 %) CNVs. The second filtration step was performed to filter out FP CNVs using two CNV quality thresholds: a low-stringency threshold (CNV quality score and CNVQ > 10; *n* = 101 CNVs) and a high-stringency threshold (CNV quality score and CNVQ > 40; *n* = 4 CNVs). Thus by removing common CNVs and using a high-stringency quality filter, we removed 99.47 % of CNVs.

To detect PIDD-causing variants, the functional and clinical annotations provided in cnvScan were examined manually. Three patients with PIDD-causing variants (two patients from the same family with a deletion in *MAGT1* and one patient with a deletion in *NCF1*) were identified from both the low- and high-stringency filtered sets. Both deletions were evaluated genetically and clinically to assess the phenotype in the respective families.

**Fig. 4:**
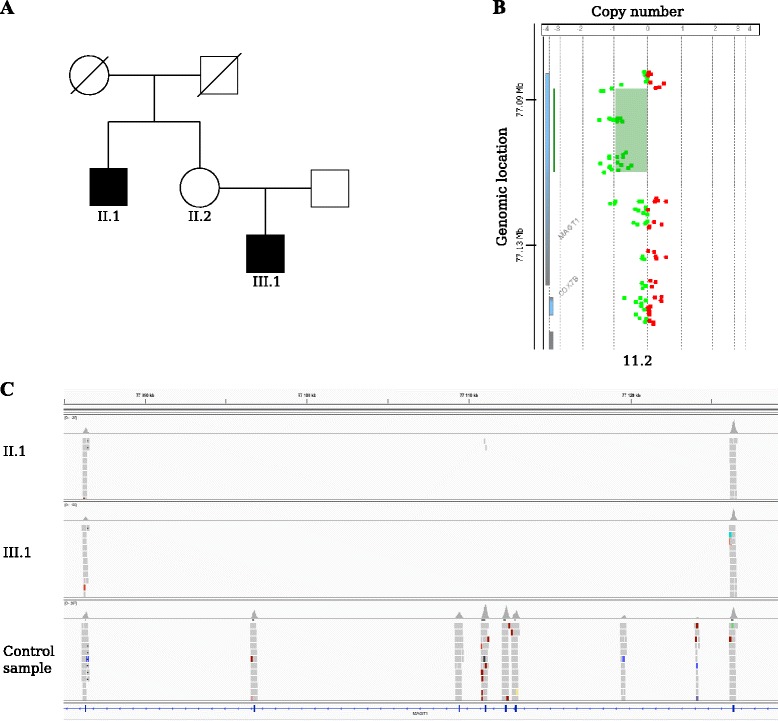
Identification of PIDD-causing variants using cnvScan. **a** Pedigree with an affected uncle and nephew (II.1 and III.1). **b** aCGH confirmation of the *MAGT1* deletion in the obligate carrier II.2. **c** IGV screenshot showing the *MAGT1* deletion (exon 3 to 8) in patient II.1 and III.1 in comparison with the normal coverage of *MAGT1* from a healthy male. The flanking exons (2 and 9) show normal coverage in II.1 and III.1, however there are no reads covering exons 3–8, indicating a deletion of the region

Defects in *MAGT1* function are known to cause X-linked immunodeficiency, and in one pedigree we detected a deletion in *MAGT1* in the proband and his uncle (III.1 and II. 2; Fig. 4a, c). The deletion was confirmed by exaCGH of the proband’s mother (II.1) who is an obligate carrier (Fig. 4b). Deletions affecting *NCF1* are know to cause recessive chronic granulomatous disease [27]. We detected a deletion in a 71 year old female with chronic granulomatous disease. The predicted deletion was confirmed by MLPA [27] to be homozygous and span the entire gene (data not shown).

We also wished to test the performance of cnvScan with the other four programs (Table 2). ExomeCopy, ExomeDepth and XHMM predictions followed by cnvScan also detected the three PIDD-causing CNVs, thus cnvScan can improve disease variant identification in other pipelines.

**Table 2:** cnvScan implementation

Program	CNV count in PIDD call set	cnvScan filtered CNV count	PIDD-causing CNV count
ExCopyDepth	769	58 (Low stringency) 4 (High stringency)	2
ExomeCopy	2782	477 (Low stringency) 180 (High stringency)	2
ExomeDepth	729	40 (Low stringency) 2 (High stringency)	2 (Low stringency) 1 (High stringency)
XHMM	151	51 (Low stringency) 49 (High stringency)	2

Low stringency parameters: CNV quality score > 10, CNVQ > 10 and not reported in public CNV datasets (Sanger high resolution CNVs, 1000 Genome CNVs and high quality DGV dataset)

High stringency parameters: CNV quality score > 40, CNVQ > 40 and not reported in public CNV datasets (Sanger high resolution CNVs, 1000 Genome CNVs and high quality DGV dataset)

CoNIFER failed to predict PIDD-causing CNVs from these exome sets

## Conclusion

Computational CNV prediction from exome sequencing data has shown high false positive CNV counts and thus had limited applicability in clinical studies. In order to improve the clinical utility of CNV prediction, we developed cnvScan to filter out false positive CNVs and to provide clinically useful annotations.

We have demonstrated that CNV quality scores (default quality score from prediction programs and CNVQ from in-house databases) can be used effectively to reduce false positive CNV counts. Functional and clinical interpretation of predicted CNVs were improved with the wide range of information provided by cnvScan annotation (gene content and functional effect, known CNVs and clinically relevant information).

In summary, cnvScan enables researchers to use different programs to predict CNVs and apply suitable filtration thresholds to remove false positives and non-disease- casing variants. This reduces the time and effort required to detect disease-causing CNVs and improves the clinical utility of exonic CNV prediction.

## Methods

### Exome capture

Exome capture was performed using the Agilent SureSelect Human All Exome capture kit v.5 (Agilent Technologies). DNA was extracted from whole-blood and 3 μg of DNA was prepared for exome sequencing according to manufacturer’s recommendations. The exome captured libraries were sequenced on an Illumina HiSeq 2500 at the Norwegian Sequencing Centre (www.sequencing.uio.no). Sequence alignment was performed with NovoAlign (v2.07.17) [28] resulting in an average coverage ~100x and 98 % of the bases covered with at least 20x. Next, initial BAM files were realigned and the base quality scores were recalibrated using GATK (v2.4) [5]. After marking duplicates with Picard (v1.74) [29], the final set of alignment data (BAM files) required for computational CNV prediction were generated.

### CNV prediction

Computational CNV prediction was performed on exomes from 17 patients with primary immunodeficiency (PIDD) using ExomeCopy [6], ExCopyDepth [4], ExomeDepth [7], CoNIFER [8] and XHMM [9]. These programs calculate a CNVQ for each prediction, but CONIFER [8] does not report this value by default. We thus changed the CoNIFER source code to report normalized singular values (SVD-ZRPKM [8]) for the left breakpoint of each predicted CNV. Moreover previous studies have shown that CoNIFER and XHMM report low CNV counts compared to other prediction programs [4, 8, 9]. Therefore CoNIFER and XHMM CNV predictions were performed with low quality thresholds (Additional file 1: Text S2) to generate a large CNV set needed for downstream analysis.

### Input data for cnvScan

The main input file required for cnvScan is the CNV results file from the prediction program (Additional file 1: Table S3).

### CNV validation (exaCGH)

Following CNV prediction, CNVs were validated using a custom CGH array (exaCGH) designed to capture exonic regions [4]. Here, exaCGH was performed using 17 DNA samples from primary immunodeficiency patients following Agilent protocol v.6.3. Agilent Genomic Workbench (v7.0) was used to call CNV regions which were detected by at least four probes (with minimum average absolute log ratio for deletion and duplication > =0.20). All array results used for calculation of FP and TP had a Derivative Log Ratio Spread (DLRS) values ranging from 0.19 to 0.42.

To identify TP and FP CNVs, we compared CNV predictions to exaCGH results. TPs are CNVs detected by both methods. FPs are CNVs detected by the prediction program but not by the array. In order to be conservative in FP identification, CNVs that have at least 4 probes in the exaCGH design were selected as the final set of FP CNVs.

Finally we calculated the cumulative TP (cTP) and cumulative FP (cFP) count to assess CNV quality scores. Quality scores of TP and FP CNVs were sorted from highest to lowest and cumulative TP and FP counts were calculated for each quality score.

For the family studies, CNVs were confirmed by either exaCGH or MLPA. MLPA was preformed by Dr Dirk Roos and Mr. Martin de Boer at the Department of Blood Cell Reasearch, Sanquin Blood Supply Organization, Amsterdam, Netherlands.

### CNV screening

CNV screening is the initial analysis performed after reading the input files. This provides metadata that describes the quality of each CNV call. The reported metadata are: CNV quality score assigned by the prediction program and CNVQ from cnvScan.

In-house database was designed to identify FP CNVs predicted due to technical artifacts in the reference exome collection. Thus, in-house database should contain CNVs predicted from the reference collection used in the prediction program. A method and script used to generate the in-house CNV database is discussed in Additional file 1: Text S1. Therefore, cnvScan users can create in-house CNV databases from reference exome collections used in their studies.

Previous studies have shown that the use of male and female samples in the same reference collection influence the CNV prediction in X and Y chromosomes [4, 9]. Thus performance evaluation of CNV screening was completed by excluding CNVs in X and Y chromosomes.

### CNV annotation

In order to interpret the functional effect and study clinical significance of the CNV, predicted CNVs were annotated with data from external databases (Table 1). Annotation process use public databases that contain CNVs detected from multiple platforms (eg. DGV CNVs) and CNVs predicted from different programs (with different length distributions [4]). Since breakpoints of these CNVs can vary depending on the original platform or computational program used, we search for at least 1 bp overlap between predicted CNV and the source dataset. Links to source datasets need for annotation process are described in wiki page in https://github.com/PubuduSaneth/cnvScan.

### CNV filtration

Following the cnvScan analysis, CNVs can be filtered to generate a set of high-quality rare CNVs. This CNV set can be further examined to identify clinically significant CNVs. Filtration is performed based on candidate gene lists (gene based filtering) and parameters reported in cnvScan analysis (variant based filtering). Separate scripts for gene and variant based filtering are available in our git repository.

cnvScan is written in python programming language and all the scripts are available via our git repository (https://github.com/PubuduSaneth/cnvScan).

### Ethical approval and consent to participate

This project has been approved by the regional ethical committee in Norway (REK: 2014/1270 Kartlegging av genetiske årsaker til primær immunsvikt og immundysregulering) and all the participants provided a formal written consent to participate in the study.

### Consent to publish

All the participants provided a formal written consent to publish.

### Availability of data and materials

Genomic data of a person is considered sensitive data under the Norwegian Personal Data Act §2, point 8 and protected under Nordic data protection laws. Therefore the PIDD patient data (discussed in the article) cannot be made available in public data repositories. However, we have provided a dataset (CNV result files and CNV database generated from 14 exomes from 1000 genomes project) to test cnvScan at our github repository (https://github.com/PubuduSaneth/cnvScan/wiki/cnvScan-implementation).

## Additional file

Additional file 1: Table S1. Statistical methods used to calculate CNV quality scores. **Table S2.** CNVQ ratio for common TP CNVs. **Table S3.** Format of the cnvScan input file. **Figure S1.** Overview of cnvScan algorithm. **Figure S2.** CNV length vs Quality score for five CNV prediction programs. **Figure S3.** GC % vs Quality score for five CNV prediction programs. **Figure S4.** Length of simple repeats internal to CNVs vs Quality score for five CNV prediction programs. **Figure S5a.** Coverage of duplications vs Quality score for five CNV prediction programs. **Figure S5b.** Coverage of deletions vs Quality score for five CNV prediction programs. **Figure S6.** TP and FP counts in the in-house CNV database. **Figure S7.** Comparison of filtration efficiency using default quality score, CNVQ, database CNV count. **Figure S8.** Filtration efficiency of XHMM. **Text S1.** In-house database creation. **Text S2.** Thresholds used in CoNIFER and XHMM predictions. (PDF 2191 kb)

## Abbreviations

aCGH: Array comparative genome hybridization
cFP: Cumulative false positive
CNVQ: Median quality score calculated from in-house CNV database
CNVs: Copy number variants
cnvScan: CNV screening and annotation
cTP: Cumulative true positive
DECIPHER (DDD): Deciphering developmental disorders
DGV: Database of genomic variants
DLRS: Derivative log ratio spread
exaCGH: exon-focussed aCGH
FDR: False discovery rate
FP: False positive
indels: Insertions, deletions
MLPA: Multiplex ligation-dependent probe amplification
PIDD: Primary immunodeficiency disorder
SNV: Single nucleotide variant
TP: True positive.
