# Supplementary material for: cnvScan: a CNV screening and annotation tool to improve the clinical utility of computational CNV prediction from exome sequencing data
Source: BMC Genomics. 2016 Jan 14;17:51. doi: 10.1186/s12864-016-2374-2 (PMC4712464; doi:10.1186/s12864-016-2374-2)
Supplement: Additional file 1: Table S1. — Statistical methods used to calculate CNV quality scores. Table S2. CNVQ ratio for common TP CNVs. Table S3. Format of the cnvScan input file. Figure S1. Overview of cnvScan algorithm. Figure S2. CNV length vs Quality score for five CNV prediction programs. Figure S3. GC % vs Quality score for five CNV prediction programs. Figure S4. Length of simple repeats internal to CNVs vs Quality score for five CNV prediction programs. Figure S5a. Coverage of duplications vs Quality score for five CNV prediction programs. Figure S5b. Coverage of deletions vs Quality score for five CNV prediction programs. Figure S6. TP and FP counts in the in-house CNV database. Figure S7. Comparison of filtration efficiency using default quality score, CNVQ, database CNV count. Figure S8. Filtration efficiency of XHMM. Text S1. In-house database creation. Text S2. Thresholds used in CoNIFER and XHMM predictions. (PDF 2191 kb) [file 12864_2016_2374_MOESM1_ESM.pdf]

Table S1 Statistical methods used to calculate CNV quality scores

Program	Description
ExomeCopy	Log of the odds ratio of predicted copy count over the normal copy count
ExomeDepth	Log of the likelihood ratio of the data for the predicted CNV over normal state
ExCopyDepth	Log of the likelihood ratio of the data for the predicted CNV over normal state
XHMM	Phred-scaled quality of not being diploid
CoNIFER	Hard threshold is used on normalized singular value decomposed-scores

Table S2 CNVQ ratio for common TP CNVs.

Program	Database CNV count	FP count	Common TP* count	CNVQ ratio
**ExCopyDepth**	3	16	5	1.3
**ExomeDepth**	-	-	-	-
**XHMM**	3	28	3	1.1
	4	27	4	1.1

CNVQ ratio = CNVQ of TP / CNVQ of FP
*Common TP: TP CNVs which were reported in high-quality DGV, 1000 genomes CNVs and Sanger CNVs

In order to assess CNVQ ratio for common TP, all the TPs reported in public databases (high-quality DGV, 1000 genomes CNVs and Sanger CNVs) were identified as common TPs and used as queries to search the in-house database. FPs used in Figure 3 were used as FP queries in this analysis. Since Figure 3 indicated ineffective CNV quality score assignment for higher database CNV counts, we calculated CNVQ ratio for database counts > 2. ExomeDepth didn't predict any common TP CNV with database count > 2. Thus, CNVQ ratio for ExomeDepth was not calculated. CNVQ ratio (1.1-1.3) for common TPs (from ExCopyDepth and XHMM) confirmed that the quality scores were not useful in differentiating common TPs and FPs.

Table S3 Format of the cnvScan input file

Column name	Example
Chromosome	1, 2, 3… X, Y
End position of the CNV	1414393
Start position of the CNV	1416351
CNV state	0-1 (deletions), >2 (duplications)
CNV quality score	5.02

Eg. First three lines of a cnvScan input file.

1	1414393	1416351	1	5.02
1	1573834	1573954	3	4.88
1	1637053	1643925	1	37.1

Figure S1 Overview of cnvScan algorithm

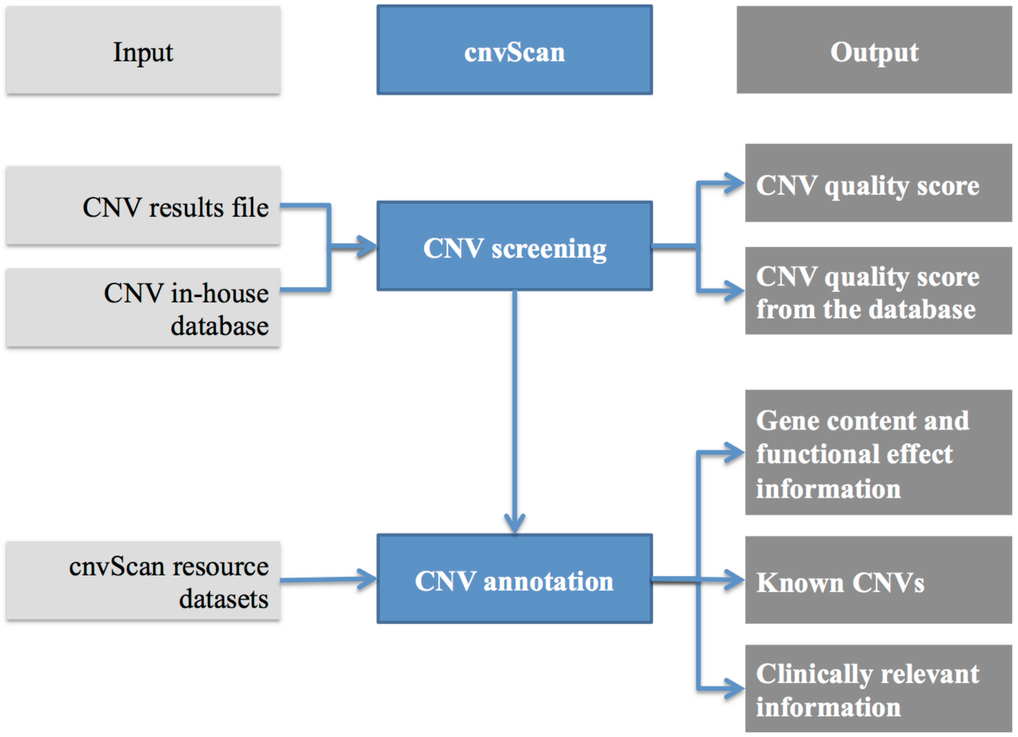

Figure S2 CNV length vs Quality score for five CNV prediction programs

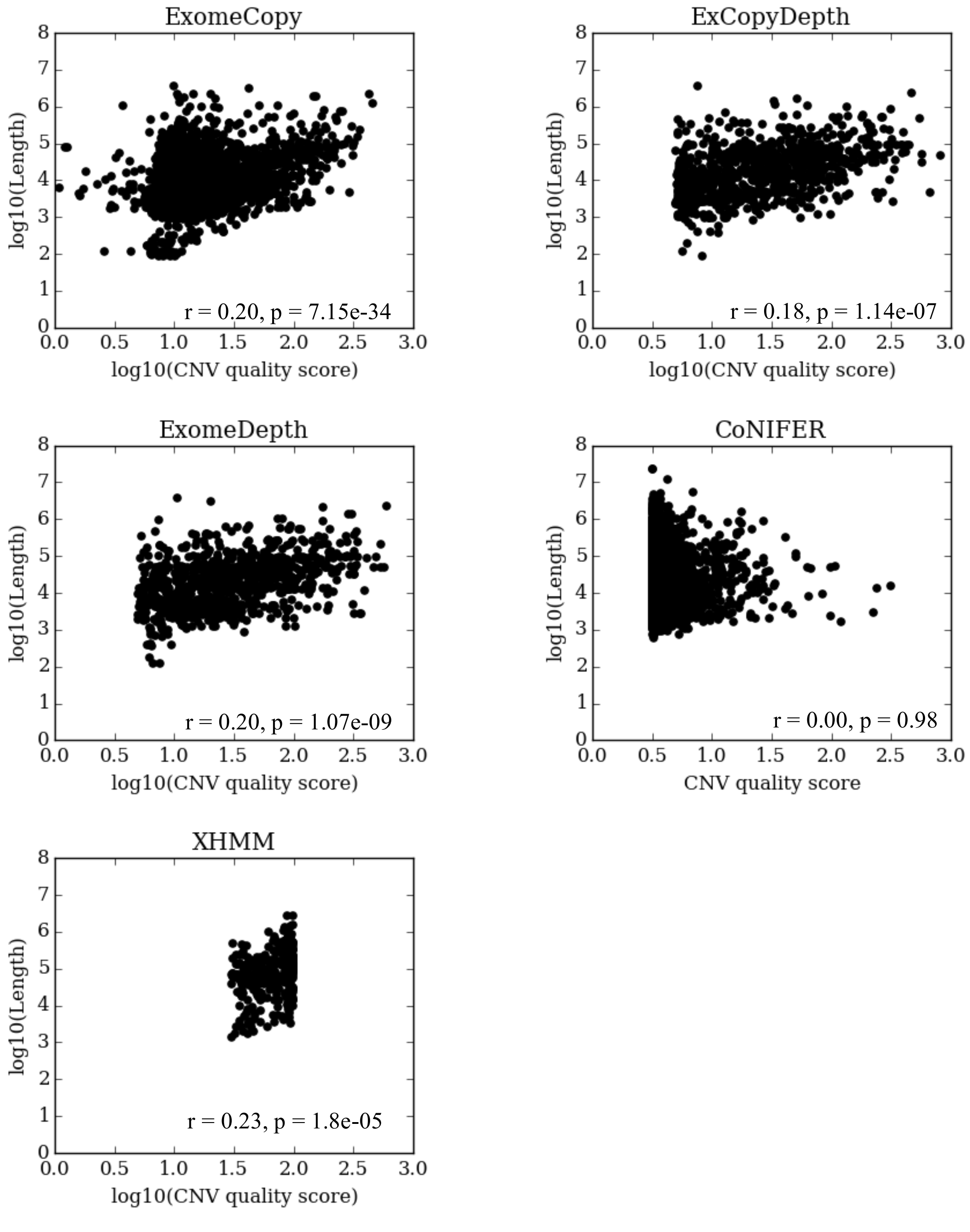

r: Pearson correlation coefficient
p: P-value

Figure S3 GC% vs Quality score for five CNV prediction programs

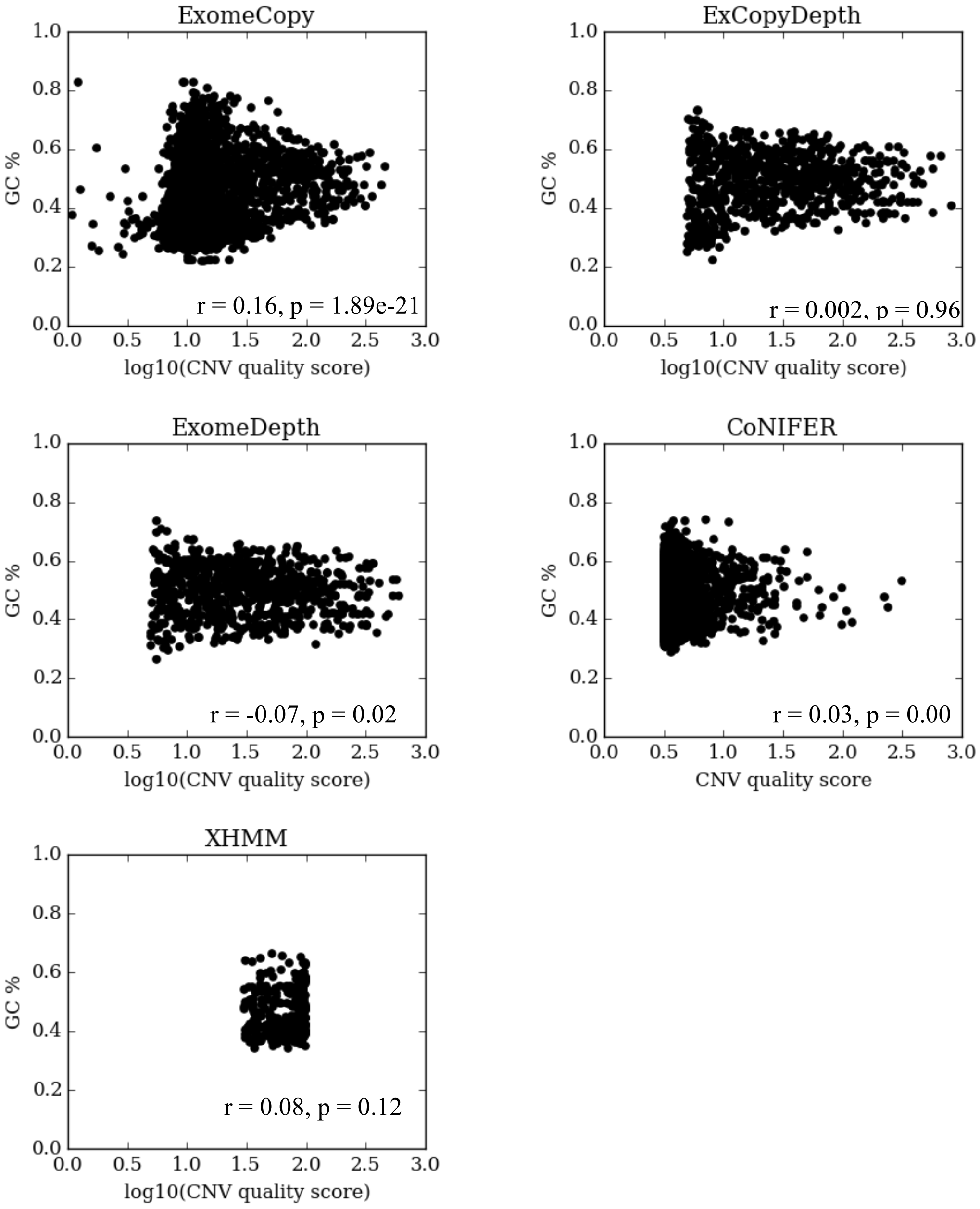

First, GC% of each exon is calculated using GATK toolkit (GCContentByInterval) and then mean GC content of exons internal to CNVs were calculated.
r: Pearson correlation coefficient
p: P-value

Figure S4 Length of simple repeats internal to CNVs vs Quality score for five CNV prediction programs

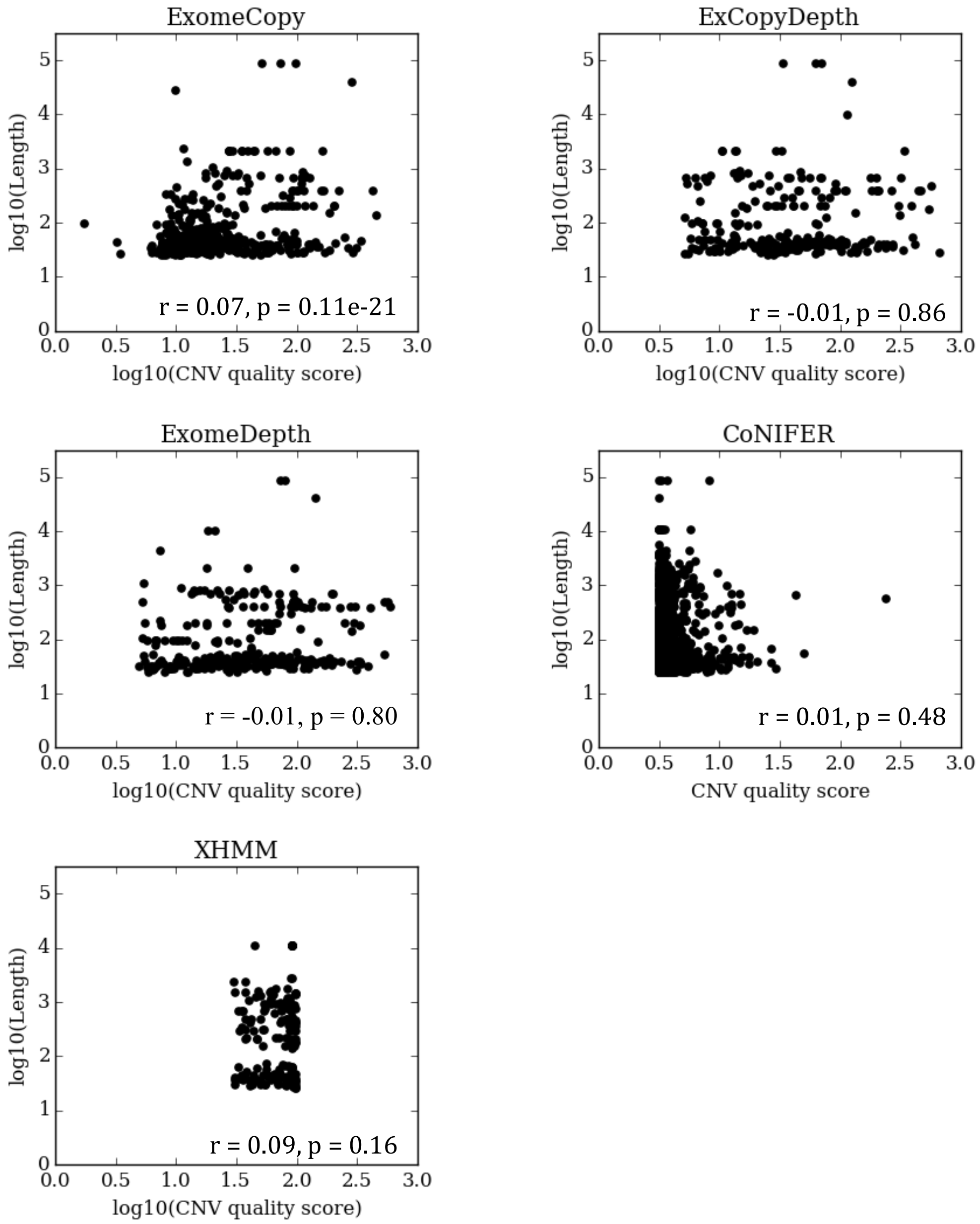

First, simple tandem repeats internal to exons were identified and then total length of simple repeats internal to each CNV was calculated.

Simple tandem repeats were obtained from: http://genome.ucsc.edu/cgi-bin/hgTables?db=hg19&hgta_group=rep&hgta_track=simpleRepeat&hgta_table=simpleRepeat&hgta_doSchema=describe+table+schema)

r: Pearson correlation coefficient

p: P-value

Figure S5a Coverage of duplications vs Quality score for five CNV prediction programs

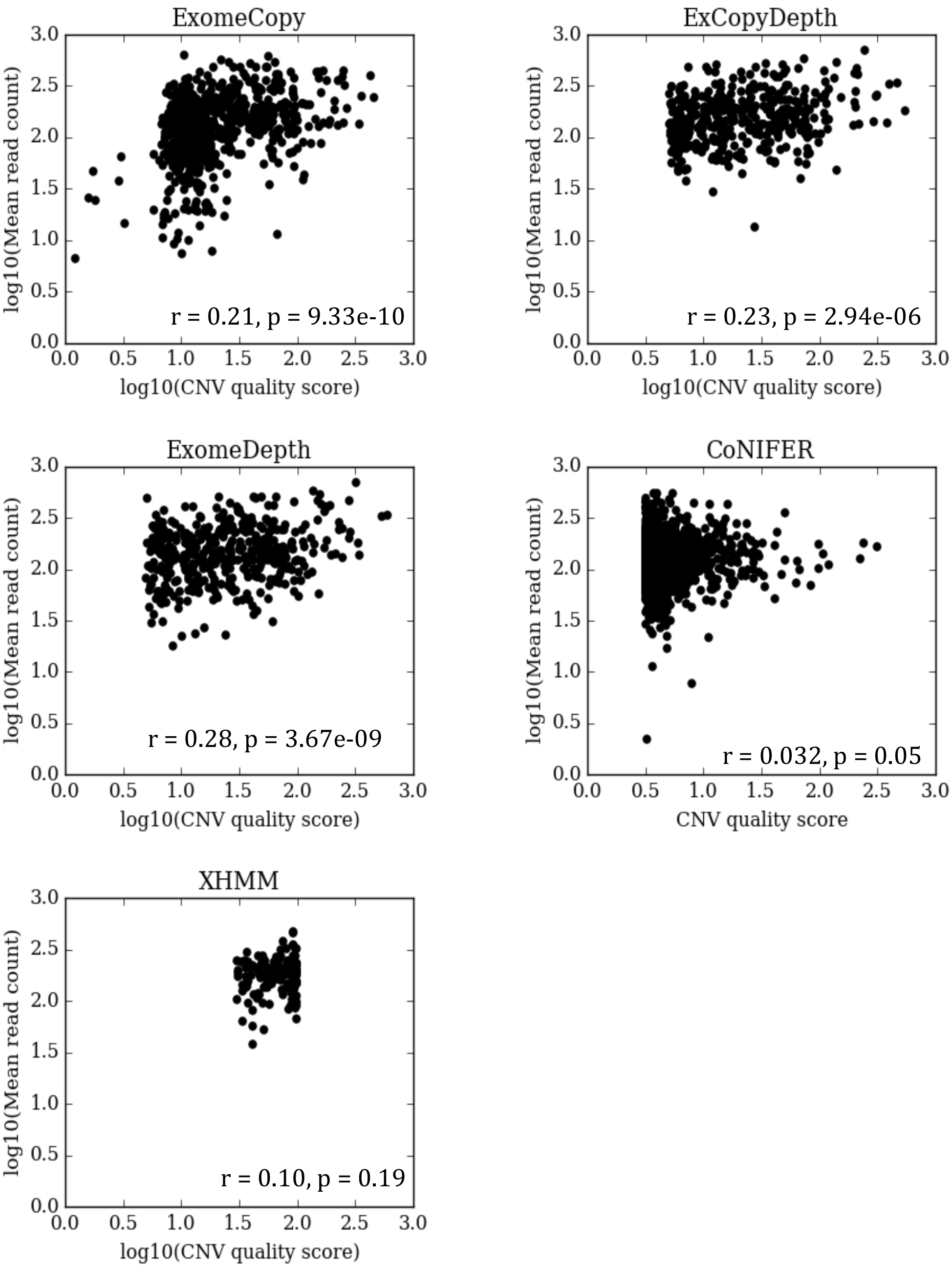

Average coverage (read depth) of CNVs were calculated using GATK toolkit (DepthOfCoverage)
r: Pearson correlation coefficient
p: P-value

Figure S5b Coverage of deletions vs Quality score for five CNV prediction programs

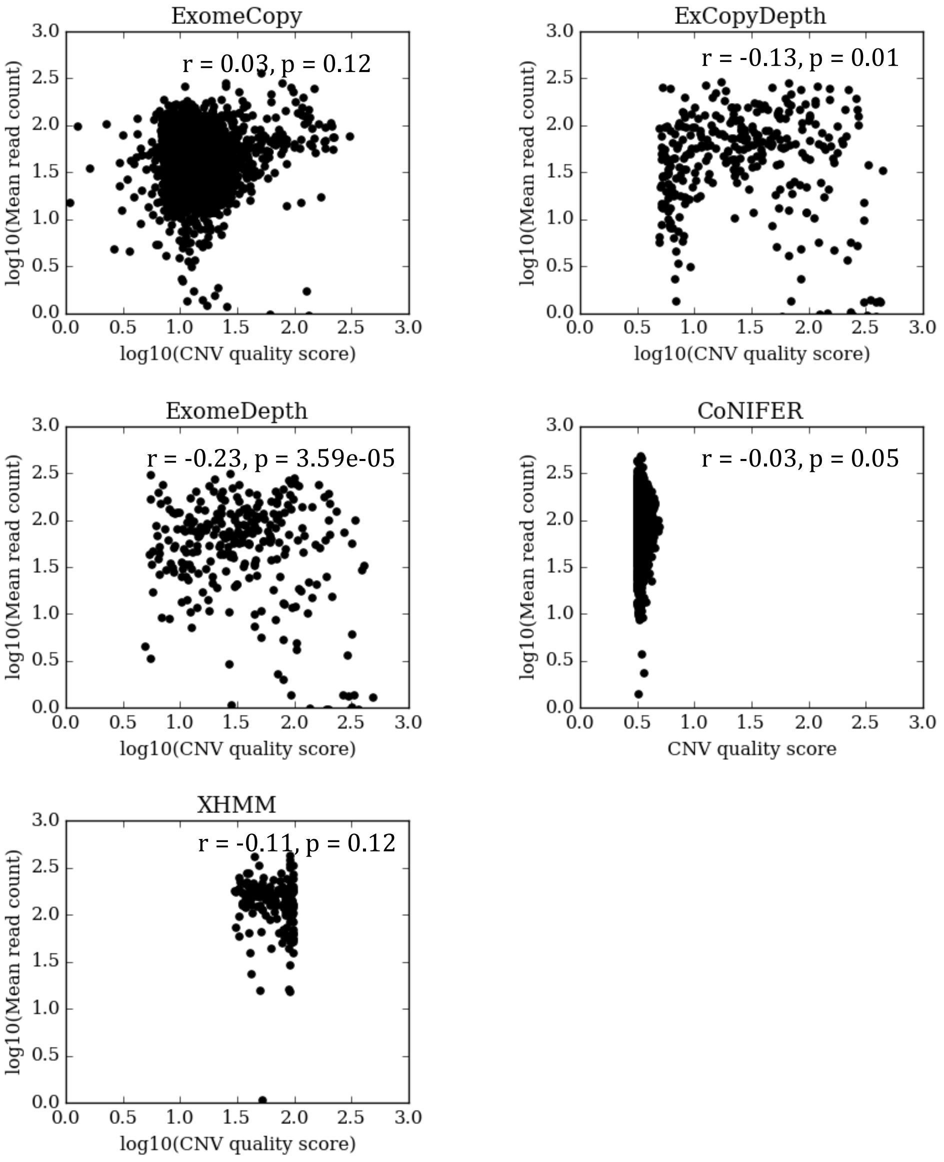

Average coverage (read depth) of CNVs were calculated using GATK toolkit (DepthOfCoverage)
r: Pearson correlation coefficient
p: P-value

Figure S6 TP and FP counts in the in-house CNV database

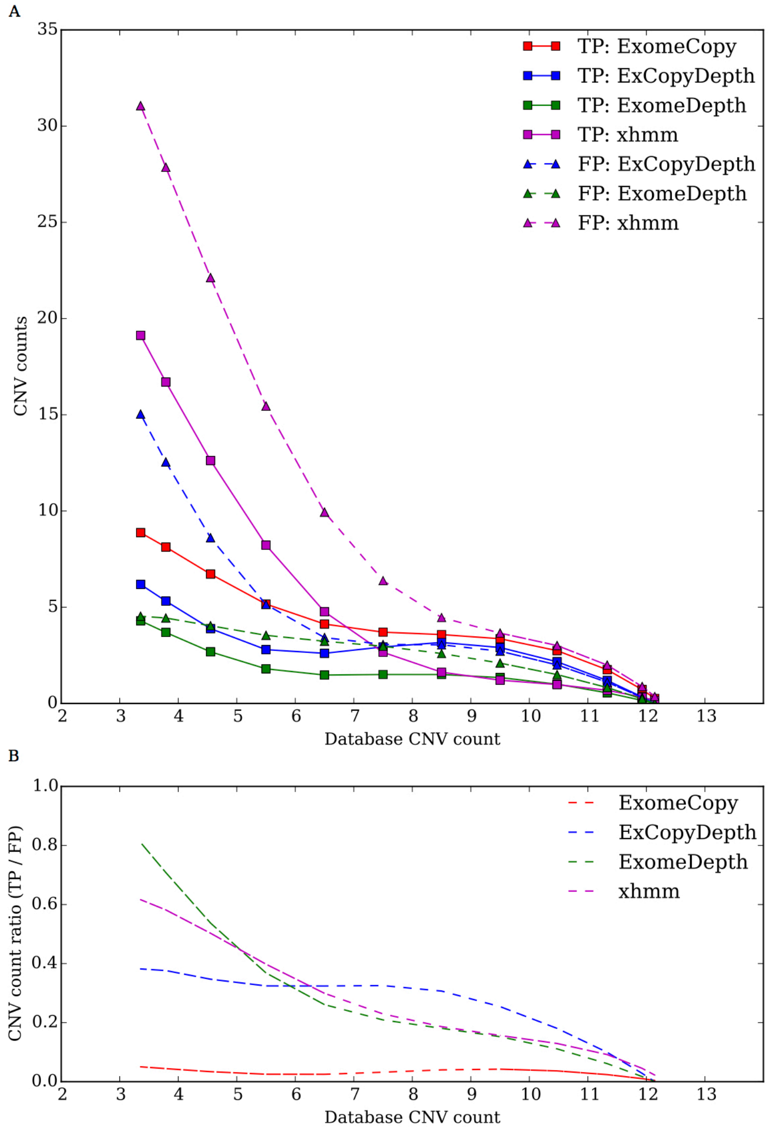

a. TP and FP CNV counts vs database counts (count distribution for TP and FP CNVs)
Database CNV count represents the number of samples in which TP or FP CNVs are predicted. FP counts were higher than the TP cunts for all the prediction programs. Thus confirms the high FP CNV prediction in these programs. FP counts of ExomeCopy were ranging from 100-200 for all the database CNV counts, thus ExomeCopy FP counts were not presented in Additional file 1: Figure 6a.

b. CNV count ratio vs Database CNV counts
CNV count ratio = TP CNV count / FP CNV count
CNV count ratio was < 1 for all the prediction programs in the entire spectrum of X-axis indicated that the FPs were overrepresented in the database.

Figure S7 Comparison of filtration efficiency using default quality score, CNVQ, database CNV count

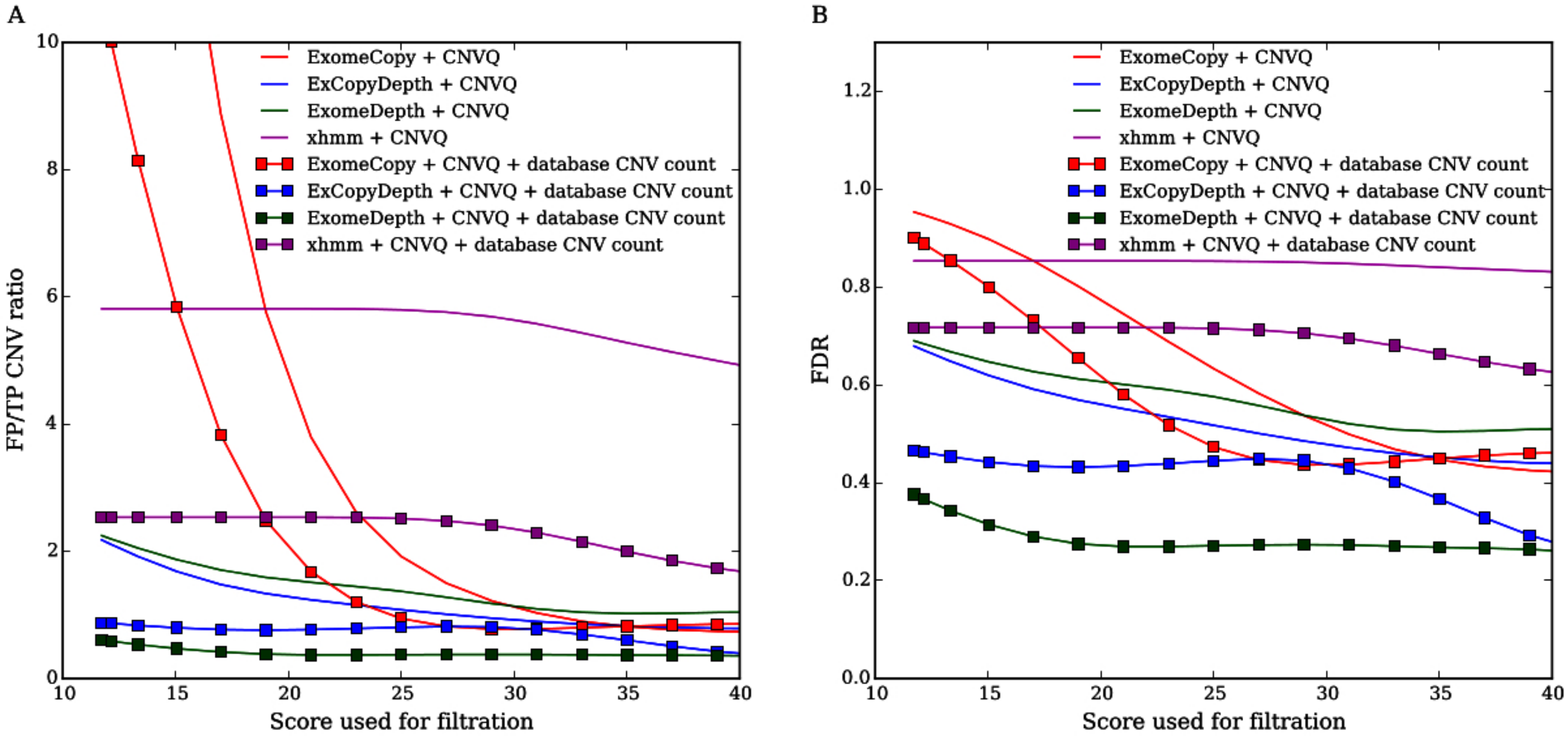

a. Comparison of cnvScan efficiency of four CNV prediction programs. Scores used for filtration: default CNV quality score from prediction programs and CNVQ from in-house database. FP/TP ratio: False positive CNV count/True positive CNV count. **b.** Comparison of the cnvScan efficiency in reducing FDR of four prediction programs (FDR of prediction programs vs cnvScan scores). FDR: False positive CNVs/(True positive CNVs + False positive CNVs).

Hard threshold on database CNV count was used (database CNV count≥5 were filtered-out) when CNVs were filtered with all three parameters. CoNIFER results were not filtered using cnvScan as CNVQ is not reported in the default state.

Figure S8 Filtration efficiency of XHMM

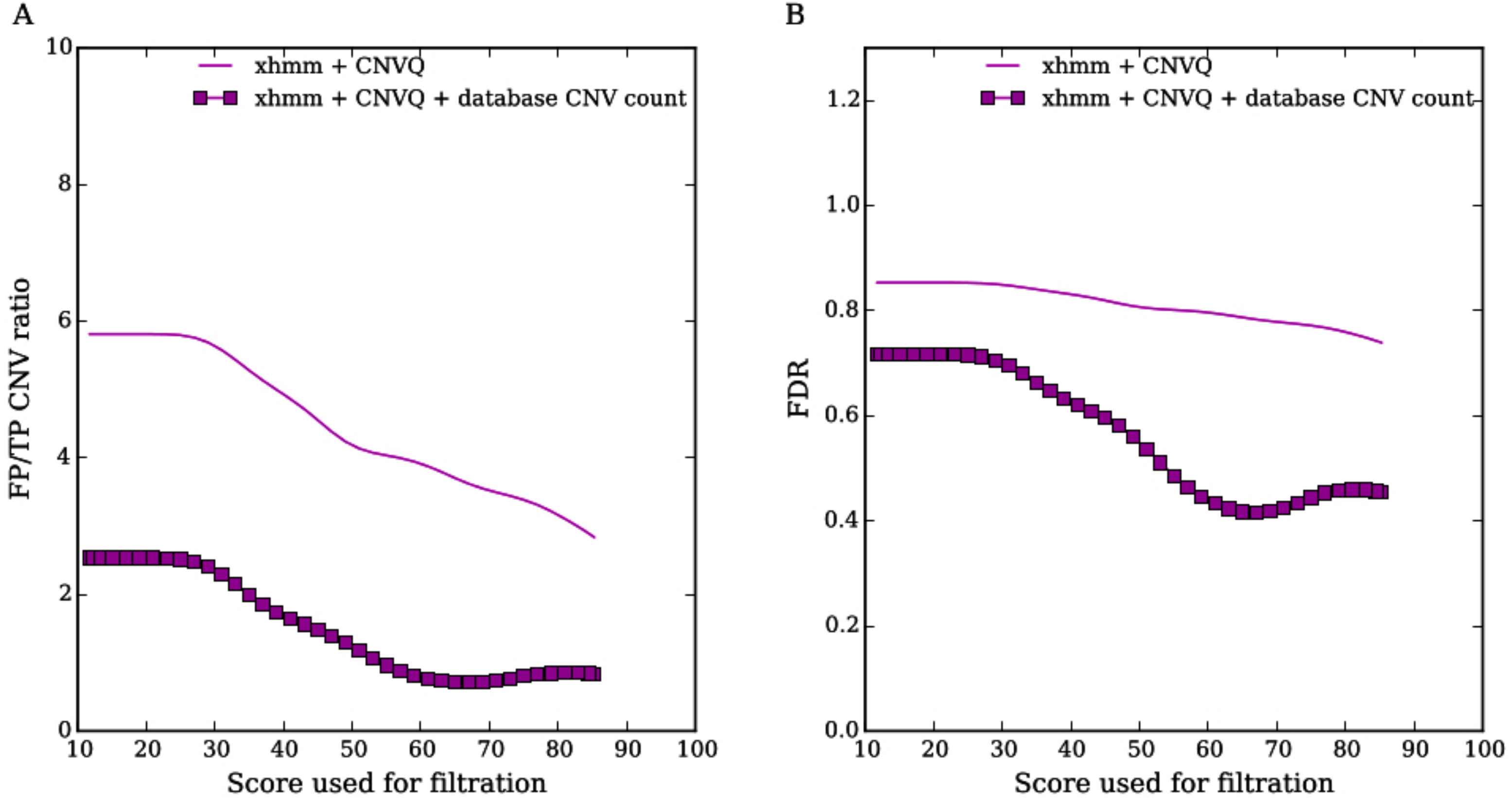

a.FP/TP CNV ratio vs cnvScan Scores. Scores used for filtration: default CNV quality score of XHMM and CNVQ from in-house database. **b.** FDR of XHMM vs cnvScan scores

Hard threshold on database CNV count (≥5) was used when CNVs were filtered with all three parameters (default CNV quality score of XHMM, CNVQ and database CNV count).

Text S1 In-house database creation.

The in-house database is an indexed flat file containing the chromosomal position of the CNV, CNV state, CNV quality score and the sample ID (sample identifier).

The following bash scripts can be used to create the database file from the cnvScan input file.

```
for f in $(find . -name "*<cnvScan input file>" -type f)
do
awk 'BEGIN{OFS="\t"}{print $0,substr(FILENAME,3,17)}' $f >> <database_file>
done

sort -k1,1 -k2n,2 -k3n,3  <database_file> >  <database_file>.sorted.bed
bgzip  <database_file>.sorted.bed
tabix -p bed  <database_file>.sorted.bed.gz
```

Note, when a sample population is processed in different batches, a separate in-house database for each batch can be created to reduce the batch effect (sequencing artefacts in different batches).

Text S2 Thresholds used in CoNIFER and XHMM predictions

CoNIFER CNV prediction:

- SVD components to remove: 5
- SVD-ZRPKM threshold: 0.5

XHMM prediction:

- Exome-wide CNV rate: 1e-08
- Mean number of targets in CNV: 6
- Mean distance between targets within CNV (in KB): 70
- Mean of DELETION z-score distribution: -2.5
- Standard deviation of DELETION z-score distribution: 1
- Mean of DIPLOID z-score distribution: 0
- Standard deviation of DIPLOID z-score distribution: 1
- Mean of DUPLICATION z-score distribution: 2.5
- Standard deviation of DUPLICATION z-score distribution: 1
